# The Debriefing Assessment in Real Time (DART) tool for simulation-based medical education

**DOI:** 10.1186/s41077-023-00248-1

**Published:** 2023-03-14

**Authors:** Kaushik Baliga, Louis P. Halamek, Sandra Warburton, Divya Mathias, Nicole K. Yamada, Janene H. Fuerch, Andrew Coggins

**Affiliations:** 1 Sydney Medical School, Westmead Hospital, Block K, Level 6, Sydney, NSW 2145 Australia; 2 Division of Neonatal and Developmental Medicine, Department of Pediatrics, Stanford University School of Medicine, Palo Alto, CA USA; 3 Simulated Learning Environment for Clinical Training (SiLECT), Westmead Hospital, Sydney, NSW 2145 Australia; 4 The Australian Institute of Medical Simulation and Innovation (AIMSi), Blacktown Hospital, Sydney, NSW 2148 Australia

**Keywords:** Simulation training, Staff development, Educational measurement, Feedback

## Abstract

**Background:**

Debriefing is crucial for enhancing learning following healthcare simulation. Various validated tools have been shown to have contextual value for assessing debriefers. The Debriefing Assessment in Real Time (DART) tool may offer an alternative or additional assessment of conversational dynamics during debriefings.

**Methods:**

This is a multi-method international study investigating reliability and validity. Enrolled raters (*n* = 12) were active simulation educators. Following tool training, the raters were asked to score a mixed sample of debriefings. Descriptive statistics are recorded, with coefficient of variation (CV%) and Cronbach’s *α* used to estimate reliability. Raters returned a detailed reflective survey following their contribution. Kane’s framework was used to construct validity arguments.

**Results:**

The 8 debriefings (*μ* = 15.4 min (*SD* 2.7)) included 45 interdisciplinary learners at various levels of training. Reliability (mean CV%) for key components was as follows: *instructor questions μ* = 14.7%, *instructor statements μ* = 34.1%, and *trainee responses μ* = 29.0%. Cronbach *α* ranged from 0.852 to 0.978 across the debriefings. Post-experience responses suggested that DARTs can highlight suboptimal practices including unqualified lecturing by debriefers.

**Conclusion:**

The DART demonstrated acceptable reliability and may have a limited role in assessment of healthcare simulation debriefing. Inherent complexity and emergent properties of debriefing practice should be accounted for when using this tool.

## Background

Effective debriefing is a key element in the learning from healthcare simulation [1]. The debriefers of simulation-based medical education (SBME) events are responsible for guidance of many participants and balancing a variety of learning needs [2]. Debriefing is viewed as a challenging skill to develop, and self-appraisal of skills may not always align with actual quality as perceived by experts [3]. As a result, the study of the availability and practical utility of debriefing assessment tools is an important consideration for healthcare simulation educators.

In most debriefings, the learners are asked to reflect on their experience and self-identify gaps in performance [4]. On occasion, we have observed that enthusiastic debriefers may make well-intended attempts to directly address perceived deficiencies using feedback. Providing suggestions and information without exploring the “why?” has the potential to stifle reflection. Exploration of the underlying thought processes leading to the various actions taken often is viewed as a characteristic of simulation debriefing that sets it apart from feedback [5]. In addition, the effectiveness of a debriefing is likely to be proficiency of the debriefer(s), but it remains unclear how to develop and assess skills [6–8].

**Fig. 1:**
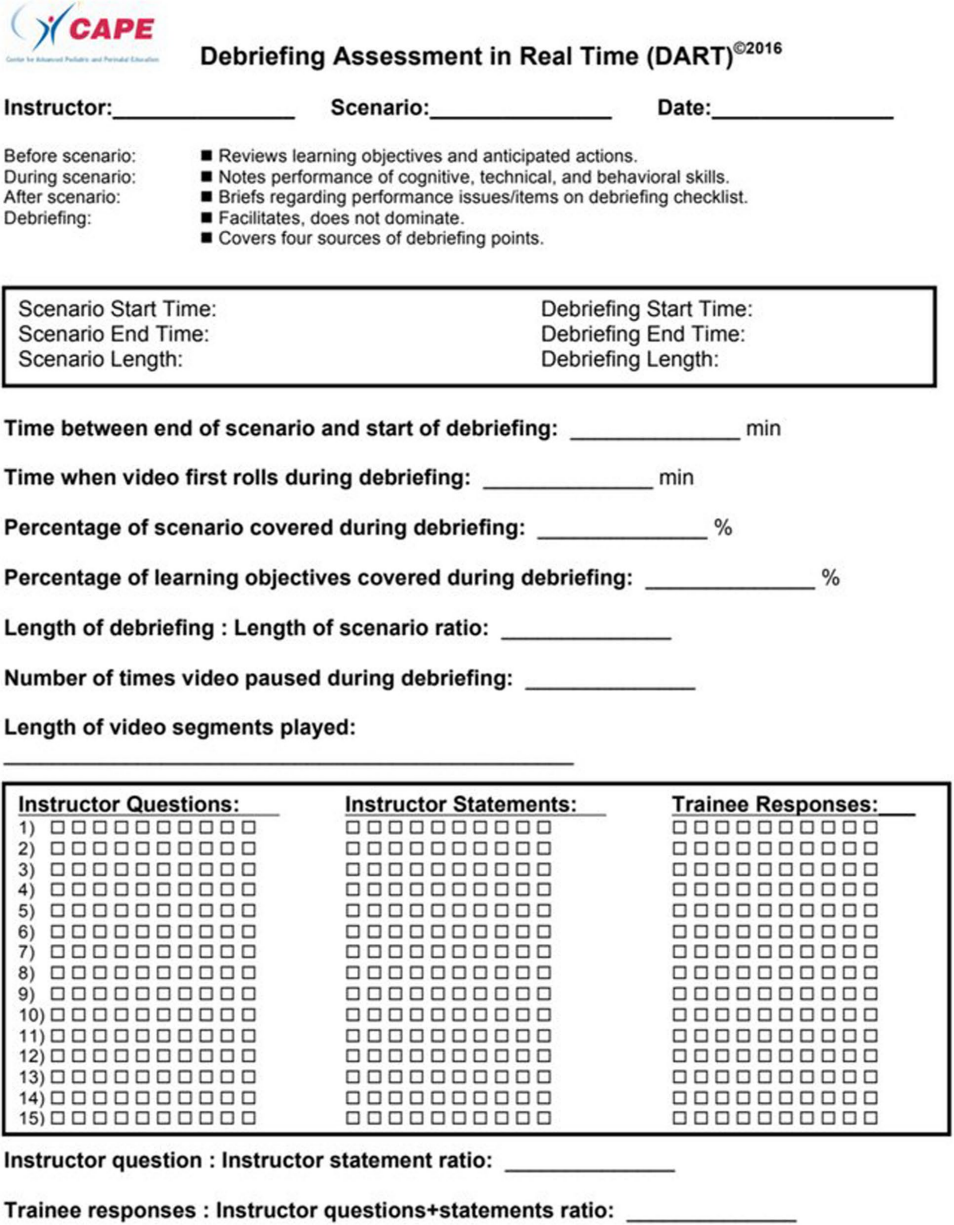
Debriefing Assessment in Real Time (DART) tool

High-quality SBME assessment instruments have previously been developed to assess these skills, the two most widely cited being the Objective Structured Assessment of Debriefing (OSAD) and the Debriefing Assessment for Simulation in Healthcare (DASH) [5, 9]. Likert scales and various domains are used with both tools requiring a qualitative assessment by either the learner(s), supervisor(s), or debriefer(s) [10]. However, in our view, a potential gap exists for additional tools that assess debriefings quantitatively and focus on the conversational dynamics. In this study, we set out to assess the effectiveness of the Debriefing Assessment in Real Time (DART) tool as an alternative or additional assessment instrument [3, 5]. DART (Fig. 1) was adapted from observing effective debriefing approaches in nonmedical industries by faculty from the Center for Advanced Pediatric and Perinatal Education (CAPE) [11]. DART purports to measure the conversational interactions between debriefers and learners using a cumulative scoring of discrete contributions. In contrast with other quantitative instruments such as DE-CODE, which was developed primarily for research, DART aims to make a real-time additional or alternative assessment for faculty development [12]. DART can be downloaded from the National Library of Medicine using an open access link from previous papers describing its use [11, 13]. In summary, this study aims to evaluate the reliability and external validity (using Kane’s framework) of the DART [14, 15].

## Methods

### Study design

The study was divided into three phases (A to C) as follows:

- *Phase A* (October 2020–March 2021) — (i) Prospective simulation participant consent, (ii) recording of consecutive debriefings, and (iii) preparation of videos for rating
- *Phase B* (March 2021–September 2021) — Video scored following training
- *Phase C* (September 2021–March 2022) — (i) Post-experience survey, (ii) videos assessed for quality by other raters (DASH), and (iii) data analysis aided by statistician

### Setting and aims

The study was a collaborative work among CAPE, Stanford (USA), and three Australian simulation centres in the Western Sydney Local Health District (WSLHD) network. In-kind resources were used with no external funding.

### Sample size

To estimate an appropriate sample size for estimating reliability and validity of the DART, a senior statistician was consulted. The advice provided suggested enrolment should include a sample of 10 debriefings with at least 8 raters. Target sampling was achieved but over a longer period than intended due to the COVID-19 pandemic.

#### Phase A — healthcare simulation debriefing video production

Debriefings are sometimes videoed (with prior consent of learners) for ongoing faculty development. Following informed consent of simulation centre attendees (see next section), we recorded a series of debriefings where practicable and universal consent was possible. To assess the DART, video assessment was chosen because live scoring with an adequate number of raters was considered impractical. Data files relating to the study were processed and stored using secure WSLHD servers.

#### Phase A — video participants

For the videos, a convenience sampling approach based on predefined criteria was used. All available videos (*n* = 8) were included to minimise selection bias. Eligible debriefings were those that included ‘professional/student (learners) participating in a healthcare simulation debriefing’ AND the ‘debriefer(s) formally trained to facilitate debriefings’. The minimum acceptable level was defined as 2 days of training. The included debriefings were to be as follows: (i) < 10-min post-event, (ii) involved ≥ 3 people, (iii) > 10 min in length. The exclusion criteria were as follows: (i) debriefer(s) not trained (in a recognised faculty development programme), (ii) refusal of individual consent from any learner(s) or debriefer(s), (iii) no availability of video recording equipment, (iv) debriefing < 10 min in length; (v) debriefings of actual clinical events or in situ simulated events; and (vi) debriefings of ‘pause and discuss’ or rapid cycle deliberate practice. Videos were recorded on a smartphone device and uploaded to a secure server.

#### Phase A — video data collection

Individuals appearing in videos and raters* were asked for demographic data including the following: (i) gender (M/F/O), (ii) current role, (iii) level of training, (iv) approximate number of years of experience, and (v) *approximate number of debriefings facilitated. Video data included the following: (i) total length (minutes); (ii) scenario topic (i.e. sepsis, cardiac arrest, asthma, acute coronary syndrome); (iii) location of simulation (A = Westmead Centre; B = Blacktown Centre; C = Auburn Centre); and (iv) number attending the debriefing.

#### Phase B — DART tool rating

DART (Fig. 1) tallies debriefing contributions including instructor questions (IQ), instructor statements (IS), and trainee responses (TR). In addition, ratios (IQ:IS and TR: IQ+IS) can be calculated. Ratios were used to quantify and assess the dynamic balance between debriefers and learners. DART tool data points (Fig. 1) not related directly to the debriefer and learner contributions (such as number of video pauses and learning objective coverage) were not collected because video playback was not utilised in the 8 debriefing samples. Raters of the videos were recruited from faculty from 6 simulation centres in two countries. Tool training consisted of a 5-min online training video [16] followed by 10 min for practice with one investigator (A. C.). Raters were asked to score the videos and return their results within 1 week.

#### Phase C — post-experience survey

DART raters were provided with a brief survey following their experience (which took around 10 min to complete). Raters were asked to ‘reflect on the experience of using of the DART tool’ and score on a Likert scale (1–7) as follows: (i) overall rating of the experience using the DART (extremely poor-excellent); (ii) overall rating of ease of using the DART (extremely difficult-extremely easy); (iii) overall opinion of usefulness of the DART for rating the quality of the debriefing (not at all useful-extremely useful); and (iv) overall opinion of usefulness of the DART as an adjunct to debriefer feedback (not at all useful-extremely useful). We asked for brief suggestions on how to improve the tool and other relevant comments that came to mind (free text response).

A conventional content analysis of text responses was completed by two investigators (K. B. and A. C.). Cross-checking between individual coders was prioritised to ensure veracity of content analysis with discussion leading to specific theme identification. The themes were presented to our wider study team and discussed in-depth on a series of online conference calls.

### Reflexivity statement

The available data was collectively assessed by our investigator group in considering the tool’s validity (and usability). The discussion presented is underwritten by prior experience, opinions, attitudes, and backgrounds of the study team. Therefore, we provide a statement to frame our collective reflexivity which is relevant to the interpretation of responses and reflections from the rater survey. The lead author K. B. is a senior medical student from Canada working closely with supervising author A. C. who is medical director of an Australian simulation centre with an interest in clinical debriefing. S. W. and D. M. are full-time simulation nurse educators in Sydney, Australia, who lead faculty development programmes. They have higher qualifications in medical education. L. H., N. Y., and J. F. are lead faculty at CAPE in the USA. They have an interest in debriefing based on collaboration with non-healthcare teams such as NASA.

### A priori plan for reliability and validity analysis

Mean, standard deviation (SD), and CV% (coefficient of variance) were used to assess DART scores provided by raters (IQ, IS, TR, IQ:IS, and TR:IQ+IS). Analysis was completed by a statistician. Cronbach *α*and coefficient of variation were then calculated to estimate the DART’s reliability (internal consistency). Three independent raters provided DASH scores for the video debriefings. Mean DART scores were compared with the mean DASH scores provided using Spearman rank correlation. In addition, the post-experience survey results (see above) and Kane’s validity framework were incorporated into our assessment of the DART [15].

## Results

Table 1 summarises the characteristics of the debriefings and participants. We included all 8 available video debriefings with a mean length of 15.4 min. Of the 45 learners included, there was a slight predominance of females (*n* = 26). The majority of learners had a medical background (*n* = 36). In terms of lead debriefers (*n* = 8) in each video, there was male predominance (*n* = 7), and most debriefers had less than 5 years of experience (*n* = 5). Of the 12 raters, there were more females (*n* = 7) than males (*n* = 5). Overall, raters had 94/96 (97.9%) rate of return. A total of 10/12 (83.3%) completed the reflective survey.

**Table 1:** Demographic characteristics of debriefings/course participants/debriefers and DASH score sum

**Video**	**Debriefing length (minutes)**	**Topic and location of simulation**	**Male (*n*/%)**	**Nursing participants**	**Medical participants**	**Debriefer gender, role, level of SBME experience**	**DASH score** **(sum of 3 reviewers)**
*1*	14.3	Sepsis (A)	2/5 (40%)	0	5 (PGY 1)	Male ED resident < 5 years	81
*2*	15.5	Sepsis (A)	4/5 (80%)	0	5 (PGY 1)	Male ED consultant 5–10 years	116
*3*	13.2	Sepsis (A)	2/5 (40%)	0	5 (PGY 1)	Male ICU resident < 5 years	80
*4*	13.0	Cardiac arrest (B)	3/8 (37.5%)	3 (PGY 1–PGY 8)	5 (PGY 1–PGY 4)	Male ED resident < 5 years	71
*5*	15.8	Seizure (A)	2/5 (40%)	0	5 (medical students)	Male ED resident < 5 years	96
*6*	14.0	Asthma (C)	2/5 (40%)	0	5 (PGY 1)	Female ICU RN < 5 years	90
*7*	15.5	Chest pain (ACS) (A)	2/6 (33.3%)	1 (PGY 5)	5 (PGY 1)	Male anaesthetist > 10 years	110
*8*	22.1	Chest pain (ACS) (A)	2/6 (33.3%)	5 (PGY 1–PGY 8)	1 (PGY 4)	Male cardiology RN > 10 years	95

Location of simulation: A, Westmead Hospital; *B*, Blacktown Hospital; C, Auburn Hospital

Tables 2 and 3 summarise the major outcomes of interest. Table 2 illustrates results for mean, standard deviation (SD), and coefficient of variation (CV%) (i.e. scoring reliability) for each variable. Table 3 illustrates the mean CV% and the calculated Cronbach α (i.e. inter-rater reliability) for each variable. Table 4 shows the quoted responses and invited reflections from the post-experience survey. Here, we report verbatim the typed responses provided by each rater.

**Table 2:** Mean, standard deviation (SD), and coefficient of variation (CV%) of video debriefing ratings

**Video**	**Instructor questions (IQ)**			**Instructor’s statements (IS)**			**Trainee responses (TR)**			**Ratio IQ:IS**			**Ratio TR:(IQ+IS)**		
	*Mean*	*SD*	*CV (%)*	*Mean*	*SD*	*CV (%)*	*Mean*	*SD*	*CV (%)*	*Mean*	*SD*	*CV (%)*	*Mean*	*SD*	*CV (%)*
*1*	29.2	4.04	13.9	87.8	22.0	25.1	40.3	5.6	13.8	0.36	0.13	35.9	0.35	0.05	14.8
*2*	29.2	3.54	12.1	66.9	22.1	33.0	60.7	20.9	34.4	0.49	0.19	39.4	0.63	0.15	23.7
*3*	23.8	2.45	10.3	64.7	24.2	37.4	59.0	19.0	32.2	0.44	0.24	54.2	0.67	0.14	20.5
*4*	17.3	3.31	19.2	59.9	20.5	34.2	44.3	12.8	28.9	0.33	0.17	50.5	0.58	0.12	21.3
*5*	21.0	3.07	14.6	86.2	36.9	42.8	39.8	13.8	34.8	0.28	0.12	42.5	0.38	0.09	23.5
*6*	16.4	1.86	11.4	21.5	8.1	37.4	50.7	18.3	36.1	0.86	0.31	36.1	1.35	0.42	31.4
*7*	18.1	3.45	19.1	54.0	17.4	32.2	49.3	15.8	32.1	0.37	0.15	40.4	0.69	0.20	29.3
*8*	35.5	5.92	16.7	105.8	32.4	30.6	85.3	16.7	19.6	0.37	0.13	36.1	0.62	0.11	17.5

**Table 3:** DART tool element Cronbach alpha

**DART element**	**Mean CV (%)**	**95% *CI***		**Cronbach** *α*
		*Lower limit*	*Upper limit*	
*IQ*	14.7%	12.3%	17.1%	0.978
*IS*	34.1%	30.3%	37.8%	0.918
*TR*	29.0%	23.3%	34.7%	0.931
*Ratio IQ:IS*	41.9%	37.0%	46.8%	0.852
*Ratio TR:(IQ+IS)*	22.8%	18.8%	26.7%	0.964

**Table 4:** Post-experience survey results

	*Overall rating of your experience using the DART tool (scales 1–7)*	*Overall rating of ease of using the DART tool (scales 1–7)*	*Overall opinion of usefulness of this tool for RATING the QUALITY of the observed debriefing (scales 1–7)*	*Overall opinion of usefulness of this tool as an adjunct to debriefer feedback (scales 1–7)*
Likert ratings mean score (SD)	5.5 (0.5)	5.5 (0.5)	5.0 (0.45)	6.1 (0.54)
	*Occurrences n (%)*	*Examples of suggestions for improvement/ relevant reflections*		
Training process	7 (28%)	• Calibration exercise prior to use of tool was helpful • A brief user guide with written examples on what constitutes a new statement and whether to consider a question/statement if repeated by same faculty would be helpful • Clarification on how to score multiple thoughts from the same speaker • Ambiguity with regard to how to score on the numbered rows in the tool • Hard to know where to split statements when scoring/tallying		
DART tool use	10 (40%)	• Have a row of tallies for each topic within the debrief • Should we rate quality of questions? • Found the tool visually easy to use with the quick tick box system • Mental requirements of paying attention took away from ability to reflect on quality of debrief, tool requires attention to detail, and it limits the number done on any 1 day • Added section for free text would be beneficial for faculty feedback		
Applicability to practice	8 (32%)	• Need to indicate what the DART scores mean • Unsure what the appropriate ratios are for effective debriefing • Not sure if scores correlate to good debriefing but could certainly help start a conversation between faculty and the tool focuses attention • Useful tool to provide objective feedback • Mixed evaluation of the debrief will be more beneficial for the debriefer; the DART tool can be recommended in conjunction with maybe the short version of the DASH tool • In a very junior group, more statements are required than a senior group, so interpretation of the result is required		

As a secondary outcome, DART was compared to an existing tool (DASH). Mean DART scores were calculated across 8 videos and compared with DASH scores from 3 raters who have received training on using DASH. Total DASH scores (×92.4; range 71–116) are presented in Table 1. These were returned for each debriefing, and rankings for total score were as follows: (i) debriefing 1 — DASH score 26 + 29 + 26 (total score 81) — rank 6; (ii) debriefing 2 — DASH score 39 + 40 + 37 (116) — rank 1; (iii) debriefing 3 — DASH scores 25 + 29 + 26 (80) — rank 7; (iv) debriefing 4 DASH score 25 + 22 + 24 (71) — rank 8; (v) debriefing 5 — DASH score 32 + 30 + 34 (96) — rank 3; (vi) debriefing 6 — DASH scores 28, 31, and 31 (90) — rank 5; (vii) debriefing 7 — DASH scores 36, 38, and 36 (110) — rank 2; and (vii) debriefing 8 — DASH scores 30, 35, and 30 (95) — rank 4. In comparing DART and DASH, we found the following correlations (Spearman): mean TR:(IQ:IS) ratio (*r* = 0.21), mean IQ:IS ratio (*r* = 0.22), mean IQ (*r* = 0.25), mean IS (*r* = 0.1), and mean TR (*r* = 0.21) suggesting poor correlation. There was good inter-rater agreement among the 3 raters of DASH scores with a Cronbach *α* of 0.958.

## Discussion

Healthcare simulation can lead to important learning opportunities, but the impact is dependent on the quality of debriefing [17, 18]. The primary aim of this study was to estimate the overall reliability and validity of the DART tool. In this discussion, we first consider the findings relating to reliability (Table 2, Table 3) and then use our experience of conducting the study and examination of all available data to construct validity arguments (Table 5). We refer to existing theories which may be of relevance, discuss the potential role of the DART tool, and discuss limitations. As a vertical theme, we consider the implications for faculty development.

**Table 5:** Kane’s framework relating to inferences on the validity of the DART tool [14, 15]

**Assessment decision(s)**	a. Determine debriefer’s approach towards facilitation (i.e. relative level of ‘guide on the side’ versus ‘sage on the stage’ behaviour exhibited during a debriefing by the facilitator) [17] b. Type of feedback to be given to debriefer(s) by co-faculty of health professions educators supervisors (i.e. DART tool focuses feedback to an observed debriefer)
**Scoring** *Are the scores provided by the DART tool appropriate to assess debriefing?*	• DART tool scale uses a cumulative tally of instructor statements, instructor questions, and trainee responses. Ratios of these cumulative scores may be calculated. The approach of using cumulative scoring was adapted by LH following experience of observing the debriefing of teams at NASA [19] • The notion of ‘lumpers’ and ‘splitters’ found when tallying instructor statements, instructor questions, and trainee responses mirrors the natural mental processes that allow for the classification of things through grouping and differentiation. What individuals ‘lump’ or ‘split’ are partially dependent on their cognitive socialisation [20] • Inter-rater reliability was investigated in this study and prior pilot study using a large number of simulation educators as raters [13] • This study occurred using videos of debriefings rather than real time limiting the analysis • The DART scale risks oversimplifying global assessment of debriefing quality in two areas as follows: (1) Assessment of the full context (how well was the facilitation of the whole simulation activity?) — this may require use of OSAD or DASH scores (2) Quality of individual questions — this may require a gestalt interpretation • Raters require an orientation to the tool to minimise error in scoring statements [13]
**Generalisation** *Are the scores observed likely to be reproducible?*	• Study site was external to that of the tool developers, and no developers evaluated the tool • DART displays reproducibility of scores [13] and Cronbach *α* > 0.85 • This appears acceptable when compared to reported reliability of tools used to assess clinical teaching [21] and entrustable professional activities • Of concern, good quality questions may be preceded by several statements when using advocacy-enquiry techniques [17]. For example, a good quality question of this sort may have 3 statements and 1 question. This in turn may significantly alter the DART scores and could explain lack of association between DASH and DART scores For novice learners, it might be appropriate to ‘provide information’ — this in turn will affect DART scores
**Extrapolation** *Do DART tool scores reflect debriefer performance?*	• Expert-novice differences not demonstrated • No evidence for individual debriefer improvement over time through use of DART • Cutrer and colleagues described master adaptive learners’ improvement over time [22]. A similar conceptual framework is described in debriefers (Cheng et al., 2020). [23] In this context, Cutrer and colleagues described informed self-assessment as important with feedback that is ‘clear, timely, specific and constructive feedback offered by trusted, credible supervisors’. These ideas would appear relevant to debriefer development with the DART tool, as well as other assessment tools aiding this process • No correlation/association was observed between DART scores and DASH scores • In other settings, simple objective data has been clearly shown to improve actual performance as follows: 1) Real-time objective audio-visual feedback of CPR performance such as chest compression depth, chest compression rate, and ventilation rate lead to improvements of those objective measures of CPR performance and improvements in the rate of ROSC [2, 23] 2) Real-time quantitative feedback in the form of mean concentric velocity displayed in front of participants leads to improvements in physical performance of strength exercises and improvements in motivation, competitiveness, and mood [24] Cutrer et al. suggested that using data can be a powerful tool to change behaviour [25]
**Implication** *What is the impact of the DART tool on debriefers?*	• Qualitative data from users (Table 4) suggests that raters are unsure how to interpret the scores • DART scores identify debriefer’s relative inclusivity and student centeredness, but scores would need to be interpreted broadly in a wider whole of simulation context by experienced simulation • DART ratios with low TR:(IQ+IS) ratios could indicate when debriefers lecture, which is a common pitfall as feedback is educator driven, instead of learner driven [17] • DART may amplify feedback to debriefers who do not elicit reflection and/or self-assessment from learners • DART may have a role in faculty development in the context of peer coaching or feedback from colleagues [7]. DART may have a role in Cheng’s conceptual framework of staged development debriefing skills over time [23]. DART may have uses at all levels of experience within this framework but will be particularly relevant in novice debriefers as to allocate attention to questions that lead to multiple responses and experienced faculty who tend to lecture during debriefings as noted above

### Reliability

Firstly, we consider the findings on reliability which build on a prior pilot study [13]. Broadly, we found that the DART tool demonstrated both between-event (i.e. between debriefings) and between-rater reliability. Cronbach’s *α*analysis of the DART components ranged from 0.852 to 0.978, suggesting an acceptable level of variation in a large pool of SBME raters. This compares favourably to scores required for high-stake assessments. When examining the mean CV% for each component, we found higher observable variances with TR (29.0%), IS (34.1%) and the IQ:IS ratio (41.9%), than the IQ (14.7%), and TR:(IQ+IS) ratio (22.8%). These findings also align with a preceding pilot [13]. The higher scoring variation in *instructor statements*could be attributable to whether the raters tended to ‘lump’ or ‘split’ their scores. ‘Lumpers’ are raters who tend to rate longer debriefer monologues as a single concept, while ‘splitters’ are raters who have the tendency to divide these contributions [20]. This may have been improved with a more detailed orientation. The main difference in this study compared to the pilot was the lower variance in IQ compared to the TR:(IQ+IS) ratio. This could suggest that a portion of the variance in TR:(IQ+IS) ratio is partially also attributable to a higher consistency in scoring IQ. Overall, the DART provided an accurate overall estimate of cumulative contributions in a debriefing, but a higher variance that was desirable was observed in the scoring of instructor statements.

### Validity

DART is an inductive tool rather than a detailed psychometric instrument but purports to measure conversational dynamics within debriefings. Collective thinking on how best to train debriefers draws on a milieu of different frameworks, so we chose to adopt Kane’s framework to discuss validity in this case [15]. Table 5 summarises our application of Kane’s framework — drawing on all the data and our reflexivity. Reviewing this table indicates some conflict in the assessments made by DART when compared to existing tools. When we draw on accepted conceptual frameworks from other disciplines [26], we argue that the tool estimates student centredness of most debriefings with the exception of very novice learners who may need a higher level of debriefer guidance. We unpack the further weaknesses of DART in the remaining discussion. We also note the lack of correlation (*r* < 0.3) of DART with DASH. This could suggest that the DART score does not give a reliable global assessment, a finding we discuss in more detail below. It is possible that a more robust conversational analysis (CA) could provide this assessment.

### Learner-centred debriefing and implications

It is generally agreed that debriefings should focus on the experience and perspectives of learners [26]. The DART aims to ensure a shift in focus and centeredness of debriefings away from the facilitator. Verbal dominance in group settings (and thereby extrapolated to debriefings) is known to be predicted by speaking time [27]. Facilitator contributions measured by the DART tool may approximate verbal dominance indicating a shift away from learner-centred reflection. Balancing the autonomy and agenda in debriefings between instructor and learners is also noted in the literature [28]. Promotion of future teamwork may not be easily achieved when a debriefer does not promote reflection [6].

We draw the reader’s attention to the key insights in Table 4 which summarises the rater experience. The key learnings that we can apply broadly to debriefing assessment include the ‘importance of training’ for any tool, steering clear from ‘ambiguity in instructions’, and the importance of ‘avoiding over-complexity’ in any tool as this can lead to distraction. Raters responding to the survey (Table 4) felt that a combination of a low number of instructor statements and higher number of questions may suggest a better debriefing, but this finding is not supported by the secondary analysis of DART compared with the DASH. Therefore, we advise caution in using the DART as a stand-alone assessment especially by inexperienced simulation faculty.

Having said this, we ask the reader to consider if they often observe lecturing by debriefers or the predominance of their contributions — and moreover, if this was recognised by the debriefer [29]. In addition, consider an occasion where the debriefer(s) may inadvertently interrupted the learners in their reflection [30, 31]. To provide evidence to the debriefer that this is suboptimal, it may be possible to use the DART scores (e.g. *we observed you made 82 instructor statements and asked 4 questions*). This information could stimulate a conversation as to how the faculty could improve for the next debriefing. The exception to this use of DART being valid would be where a significant performance gap is identified among the majority of learners by the debriefer. If this is clearly apparent to the debriefer(s) at an early stage, it may be entirely appropriate to provide information (lecture) to address the gap [17]. We have experienced this scenario in debriefing of novice learners such as medical students. We are also duly reminded of the emergent properties of simulation debriefing and the need to use gestalt and common sense in interpreting the results of any assessment instrument.

A further caveat is that simply asking lots of questions does not necessarily result in a high-quality debriefing. Poor quality questions may confuse or even harm learners resulting in both uninformative answers and a breakdown of trust [32]. Likewise, as noted above, a high number of debriefer statements and guidance might be appropriate with novice learners. An accurate evaluation of the quality questions could be possible using CA methods, but it may require a video or multi-rater analysis [33]. In our view, questions that elicit multiple responses from multiple learners are most likely to be valuable [29]. The lack of assessment of question quality in DART is problematic for broad validity — but could be overcome by concurrent use of existing tools, writing down quotes, video playback, or the use of relational diagrams [10, 34]. We recall the latter being used in problem-based learning (PBL) facilitator training in the mid-2000s and note it could now have a future application for simulation faculty development.

### Usability

Table 4 gives insight into the DART’s usability. We report encouraging results for ‘ease of use’ and *‘*overall use’ on a Likert scale (*μ*= 5.5/7). These results contrasted from a statement provided by one user ‘[sic] concentration of using the tool took away from observing the debriefing’. This may reflect that the need to reduce cognitive load does remain an issue in the delivery of simulation [3].

Regular use in a busy simulation setting is favoured by the design being a single page tool with minimal training required. A single expereinced faculty member could score the DART and provide peer feedback. Other more time-consuming tools may not allow sufficient time for immediate peer-feedback [10]. We will make modified-free infographic form of DART tool available at www.emergencypedia.com/CAPE) [7].

Responses from the reflective survey identified three recurrent themes — ‘training’, ‘tool-use’, and ‘applicability’. Regarding training, survey respondents described the need for more clarification on how to score statements which we have discussed above in detail. One respondent stated that ‘calibration exercises were helpful’ but expressed that they would have benefitted from written examples on how to score. Secondly, an ‘easy to use’ was reported by 3/10 respondents, but as noted above, there was concern about the cognitive load of using the tool. Thirdly, regarding application, users were uncertain as to how DART scores can measure the quality of a debriefing (Table 4). Overall, there was a positive response towards the DART by the users with a mean score of 5.5 (0.45 SD) across the survey items, possibly indicating an interest among the users in adapting the tool to existing faculty development approaches in their setting.

### Limitations

While this study was prospectively conducted, observational data is prone to bias and confounders. We note that inferences drawn from this data set are at risk of being affected by bias and advice caution in extrapolation. Furthermore, all participants provided written consent to being filmed so a *Hawthorne effect* may have applied to their behaviour. Moreover, the use of a 7-point Likert-scale in our post-experience survey allows for subjectivity and may introduce variation in scoring.

There are also noticeable differences in the debriefing culture between CAPE in the USA, where DART was initially conceived, versus the adult simulation setting in Australia where the tool was tested (Table 5). For example, the ‘advocacy with inquiry’ (AI) approach is commonly used in the WSLHD centres [35]. As taught by proponents of AI, we offer an example of how it affects the DART scores: ‘(i) Let’s talk about (Statement 1); (ii) I noticed (Statement 2); (iii) I think (Statement 3); and (iv) I wonder (Question 1))’. Therefore, with AI being used, we would expect to observe a paradoxical high statement to question ratio in good debriefings. This phenomenon might also explain the lack of association between DART and DASH we discussed above [4].

In regard to culture, the debriefing techniques used at CAPE, unlike AI, eschews debriefer opinion and emphasises focusing on the experience of the learners. Promoting discussion with questions is favoured over sharing of observations [32]. The rationale for this is two-fold: (i) learners (especially skilled ones) typically require little guidance in discussing the details of a well-designed scenario based on learning objectives appropriate to their level of experience, and (ii) input provided by the debriefer may unintentionally sway trainee discussion in a direction away from with what the learners view as important to their learning and usual context.

### Example of DART utilisation in practice

The DART may be used to initiate feedback to a debriefer as follows: ‘So I noticed you made a 105 statements, used 7 questions, and had 18 responses from learners – can we go through these numbers and try to make sense of them in order to improve our next debriefing?’ This opening could be followed by a discussion of the relative student centredness of the debriefing as well as what might change for a next attempt. ‘It sounds as if next time you would want to ask more quality questions and make less statements about the medical expertise issues – perhaps we can tweak the scenario slightly to support that happening. What do you think?’

## Conclusions

In this study, we found evidence of reliability adding to work in a previous pilot study [13] and explored the validity and limitations of the DART. Questions remain regarding the tool’s validity and best uses in the complex area of faculty development. However, more broadly, the use of the DART and other quantitative tools for feedback to debriefers appears to be worthy of further exploration in future studies in a variety of learning environments.

## Data Availability

All data generated or analysed during this study are included in this published article. SiLECT centre data is available on request from andrew.coggins@health.nsw.gov.au

## Abbreviations

AI: Advocacy-inquiry
AIMSi: Australian Institute of Medical Simulation and Innovation
CAPE: Center for Advanced Pediatric and Perinatal Education
CV%: Coefficient of variation
DART: Debriefing Assessment in Real Time
DASH: Debriefing Assessment for Simulation in Healthcare
HREC: Human research and ethics committee
IQ: Instructor questions
IS: Instructor statements
NASA: National Aeronautics and Space Administration
OSAD: Objective Structured Assessment of Debriefing
SBME: Simulation-based medical education
SHORT: Simulation in Healthcare retrOaction Rating Tool
SiLECT: Simulated Learning Environment for Clinical Training
TR: Trainee responses
WSLHD: Western Sydney Local Health District
